# Dental erosion, prevalence and risk factors among a group of adolescents in Stockholm County

**DOI:** 10.1007/s40368-017-0317-5

**Published:** 2018-01-11

**Authors:** M. Skalsky Jarkander, M. Grindefjord, K. Carlstedt

**Affiliations:** 1 Department of Pediatric Dentistry, Public Dental Service, Stockholm, Sweden; 2 Divisions of Pediatric Dentistry, Department of Dental Medicine, Karolinska Institutet, Solna, Sweden; 3 Centre for Pediatric Oral Health Research, Stockholm, Sweden; 4 Faculty of Health Sciences, Institute of Clinical Odontology, University of Tromsö, Tromsö, Norway

**Keywords:** Dental erosion, Prevalence, Risk factors, Adolescents

## Abstract

**Aims:**

To investigate the prevalence and risk factors of dental erosion (DE) among a group of adolescents in Stockholm County.

**Materials and methods:**

This cross sectional cohort study was conducted at three clinics of the Public Dental Service in Stockholm County. Fifteen and 17 year old adolescents (1335) who scheduled their regular dental health examination were asked to participate. After drop-outs a sample of 1071 individuals, 547 males and 524 females were enrolled in the study. Presence of erosive wear was diagnosed (yes/no) on marker teeth by trained dentists/dental hygienists and photographs were taken. The adolescents answered a questionnaire regarding oral symptoms, dietary and behavioural factors. Two calibrated specialist dentists performed evaluation of the photographs for severity of DE using a modified version of the Simplified Erosion Partial Recording System (SEPRS).

**Results:**

DE was clinically diagnosed in 28.3% of 15 years old and 34.3% of 17 years old. Severe erosive wear (grade 3 and 4 according to SEPRS) was found in 18.3% of the adolescents based upon the intra-oral photographs. DE was more prevalent and severe among males than females. Clinically diagnosed erosive lesions correlated significantly with soft drink consumption (p < 0.001), the use of juice or sport drinks as a thirst quencher after exercise (p = 0.006) and tooth hypersensitivity when eating and drinking (p = 0.012). Furthermore, self-assessed gastric reflux was a factor strongly associated with DE (p < 0.001).

**Conclusion:**

The study indicated that DE was common among adolescents in Stockholm County and associated with both internal and external risk factors.

## Introduction

Dental erosion (DE) is defined as an irreversible loss of dental hard tissue caused by a chemical process that does not involve bacteria (Lussi and Ganss 2014). It is a multifactorial condition of growing concern that often occurs together with other forms of tooth wear, such as attrition caused by contact between teeth, and abrasion caused by any material with abrasive effect (Holbrook et al. 2003).

The aetiological factors of DE can be divided into ‘external’ and ‘internal’ causes. External causes include acid influence through food intake, certain medications, and work-related factors. A lifestyle with a diet containing more fruits and vegetables, sports drinks during exercise but especially an increased intake of fruit juices and soft drinks are the main exogenous causes of DE among today’s children and that both a healthy and unhealthy lifestyle can contribute to susceptibility for erosive wear (Johansson et al. 2012a; Mulic et al. 2012; Isaksson et al. 2013; Hasselkvist et al. 2014). Internal causes including diseases that cause acidic stomach contents to reach the oral cavity such as gastroesophageal reflux disease (GERD) (Wilder-Smith et al. 2015) or eating disorders with repeated vomiting (Lussi et al. 2006; Otsu et al. 2014). Reflux disease can be present without subjective symptoms, so-called ‘silent reflux’, and should always be suspected in idiopathic DE. Johansson and co-workers (2012b) showed in their study that “DE is about 8.5 times more common in patients with eating disorders”.

The global prevalence of DE in children and adolescents shows great variation with rates ranging from 7.2% (Vargas-Ferreira et al. 2011) to 95% (Al-Majed et al. 2002). A systematic review estimated an overall worldwide prevalence of tooth erosion to be 30% in permanent teeth of children and adolescents aged 8–19 years (Salas et al. 2015a). In European countries 24% (Truin et al. 2005) to 64% (Dugmore and Rock 2004a) of young people are reported to have dental erosive wear and corresponding figures for Scandinavian countries are 12–75% (Hasselkvist et al. 2010; Isaksson et al. 2013; Mulic et al. 2013; Sövik et al. 2014).

The wide prevalence range found in these studies may be explained by a variety of factors such as non-homogeneous groups of divergent ages, different examination standards, diagnostic criteria, endpoints and also groups from various social areas. The clinical index of choice for DE detection also plays a very important role in this variability (Muller-Bolla et al. 2015).

DE seems to increase with age, and to become more severe over time. In a cohort of 12 years old, a longitudinal study from UK reported new or more advanced lesions in 27% of the children over the 2 years of the study (Dugmore and Rock 2003). Another 3 year longitudinal study conducted in The Netherlands among a cohort of 10-12 years old, showed a steady progression of DE in children with erosive wear (El Aidi et al. 2011).

The relationship between the prevalence of DE and socio-economic status is contradictory. Several studies have claimed that children with low socio-economic status have a higher prevalence of DE (Al-Dlaigan et al. 2001; Dugmore and Rock 2003; El Aidi et al. 2010) while other research groups reported the opposite (van Rijkom et al. 2002; Bardsley et al. 2004; Luo et al. 2005).

The fact that DE seems to be an increasing problem in children and adolescents and may cause extensive loss of tooth substance that require complicated and expensive restorative treatments, makes it essential for clinicians to pay attention to this condition. An early diagnosis provides opportunities to influence and change eating and drinking habits, to detect medical causes of DE (Carvalho et al. 2014; Peutzfeldt et al. 2014) and to be able to implement necessary preventive measures. It is also of great importance to investigate the prevalence and severity of DE in the population to raise awareness and focus among clinicians and other health care professionals on this condition.

There is a lack of studies, evaluating the prevalence of DE in Sweden and no study regarding DE has been carried out in the region of the capital of Sweden. Hence, the aims of this study was to investigate the prevalence and risk factors of DE among a group of adolescents in Stockholm County.

## Materials and methods

The present study was conducted during 2010–2011 and designed as a cross sectional cohort study. Three clinics in Stockholm County with populations from different socioeconomic levels that included urban and suburban areas were selected as being representative of the socio-economic levels of the population in this part of Sweden. In Stockholm County there are about 22,000 15 and 17 years old respectively and approximately 75% are patients in the Public Dental Service. From the computerised recall system, 1335 adolescents (15 and 17 years of age) that were going to schedule their regular dental health examination were selected in consecutive order.

The present study was approved by the Regional Ethical Review Board in Stockholm, Sweden (Dnr: 2010/1836-31/3). All procedures performed in this study involving humans were in accordance with the ethical standards of the institutional and/or national research committee and with the 1964 Helsinki declaration and its later amendments or comparable ethical standards. Swedish adolescents 15 years of age are allowed to decide themselves whether to participate and all subjects were informed of their right to resign before or during the study. Informed consent was obtained from all individuals included in the study.

### Study population

**Fig. 1:**
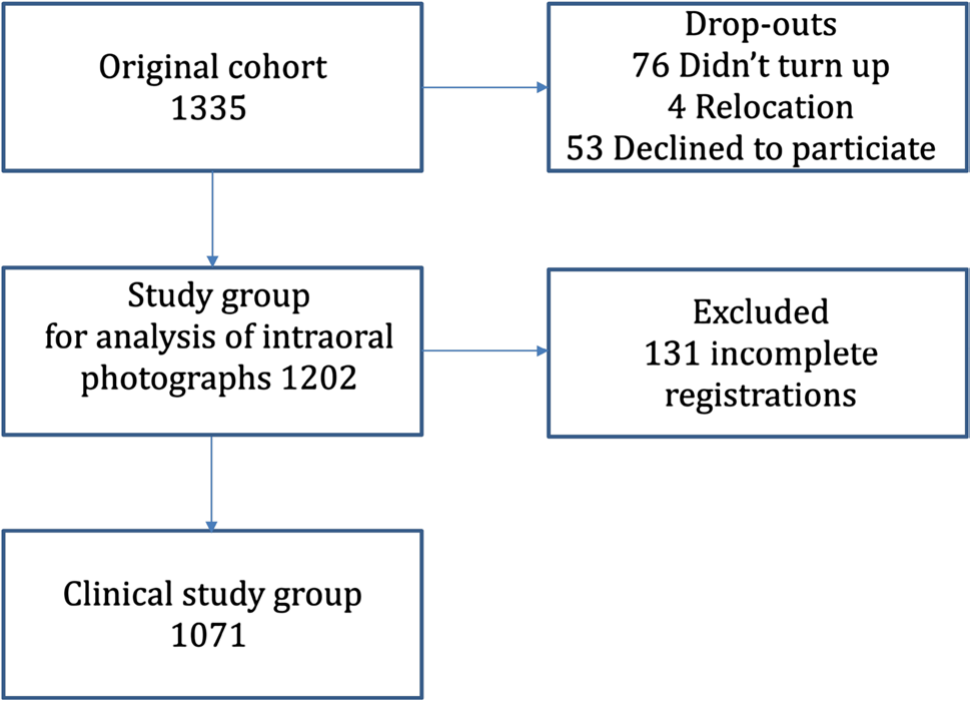
Study population and number of subjects in each phase of the study

Of the original cohort of 1335 individuals, 547 males and 524 females (n = 1071) were enrolled in the study. In Brommaplan there were 166 15 years olds and 174 17 years old, in Sollentuna 195 and 201 and in Södertälje 159 and 176 respectively. The reasons for non-participation were absence from examination (n = 76), relocation (n = 4) and decline to participate (n = 53). One hundred thirty-one individuals were excluded because of incomplete registrations concerning the presence of DE (Brommaplan n = 29, Sollentuna n = 10 and Södertälje n = 92). In the second part of the study when severity of DE was graded on the photographs taken at the regular dental health examination; the study group consisted of 1202 patients (Fig. 1).

### Informational meetings and the dental team

Participating dentists, dental hygienists and dental nurses at the three selected clinics attended an informational session and a lecture on dental erosive wear by the main author (MSJ) prior to the data collection period. They were also trained regarding how to perform clinical photographs in an accurate way, with practical exercises (MSJ). A book with pictures of DE was distributed to the clinicians as reference material (Johansson and Carlsson 2006).

### Clinical examination and questionnaire

The regular dental health examination followed a routine protocol and radiographs were taken for individual reasons. In addition, the presence of erosive wear on maxillary incisors palatally and mandibular first molars occlusally, was diagnosed by a dentist or dental hygienist at the clinic. The examination was carried out in dental clinics with modern equipment and optimal lighting and teeth were dried with compressed air prior to the clinical evaluation. Teeth with extensive caries, restorations or with fixed appliances were excluded from the screening of DE. Three photographs were taken of the marker teeth for erosive wear. One was taken of the maxillary incisors in a palatal view and one of each mandibular first molar in an occlusal view.

All subjects completed a questionnaire with an extended history regarding tooth sensitivity, oral hygiene habits, consumption of erosive drinks (carbonated, juice, sport drinks) as well as questions about lifestyle factors (computer proficiency, training, etc.) (Table 1). The photographs and questionnaires were coded at each clinic and transferred to the manager, Department of Paediatric Dentistry, Eastman Institute.

**Table 1:** Questionnaire comprising 19 questions

1. Hypersensitivity of teeth	Yes or no
2. If symptoms, when occurring	When brushing teeth or when consuming food and/or drink
3. Consumption of Coca-Cola or other soft-drinks	Several times a day, several times a week, more seldom
4. Consumption of juice or sport drinks	Several times a day, several times a week, more seldom
5. Consumption of juice at breakfast	Yes or no
6. Juice or sport drink as thirst quencher after exercise	Yes or no
7. Consumption of fruit	More than three times a day, more seldom
8. Consumption of apples	More than three times a day, more seldom
9. Consumption of citrus fruits	More than three times a day, more seldom
10. Computing	More than 3 h a day, less than 3 h a day, not every day
11. Exercise frequency	Every day, several times a week, more seldom
12. Self-assessed gastric reflux	Yes or no
13. If yes, how often	Daily, several times a week
14. Intake of fluoride tablets	Yes or no
15. Tooth brushing frequency	More than two times a day, two times a day, more seldom
16. Tooth brushing before and/or after breakfast	Before breakfast, after breakfast, before and after breakfast
17. Use of chewing gum	Daily, once a week, more seldom
18. Use of mouthwash	Yes or no
19. If yes, what kind of mouthwash	With fluoride, without fluoride

### Diagnostic criteria for severity of DE

Maxillary incisors and mandibular first molars were chosen because these teeth are known to be highly susceptible to DE. It has also been shown that using marker teeth for DE yields savings in time and reliable results. Hasselkvist and co-workers showed that a Simplified Erosion Partial Recording System (SEPRS) using four permanent surfaces 11, 21 palatal and 36, 46 occlusal gave excellent sensitivity and specificity in relation to scoring of all maxillary canines/incisors and first permanent molars (Johansson et al. 1996; Hasselkvist et al. 2010).

The severity of DE on the palatal surfaces of 12–22, diagnosed via clinical photographs, was graded on a 5-point scale according to the erosion index developed by Johansson et al. (1996) (Table 2). The severity of molar cuppings on the occlusal surfaces of 36 and 46, diagnosed via clinical photographs, were recorded according to a separate scale, constructed by Hasselkvist et al. (2010) (Table 3). The index used in this study was a modified version of the SEPRS. It has the same grading but registration is performed on six surfaces instead of four, 12–22 palatal view and 36, 46 occlusal view. The two scales for incisors and molars were combined based on the highest erosion grades scored on palatal surfaces on anterior teeth and occlusal surfaces on madibular first molars. Erosion scores were: no erosion (score 0), mild erosion (score 1), moderate erosion (score 2), severe erosion (score 3) and very severe erosion (score 4). The highest scores from the recording of maxillary anterior teeth and mandibular first molars were used to determine the total erosion score at an individual level. Qualifying for score 4 meant that there was at least one tooth surface of the maxillary anterior teeth graded 4 (Table 2) or a molar occlusal surface with cupping score 4 (Table 3).

**Table 2:** Ordinal scale used for grading severity of dental erosion on lingual surfaces of maxillary incisors (Johansson et al. 1996)

Grade	Criteria
0	No visible changes, developmental structures remain, macro-morphology intact
1	Smoothened enamel, developmental structures have totally or partially vanished. Enamel surface is shiny, matt, irregular, ‘melted’, rounded or flat, macro-morphology generally intact
2	Enamel surface as described in grade 1. Macro-morphology clearly changed, faceting or concavity formation within the enamel, no dentinal exposure
3	Enamel surface as described in grades 1 and 2. Macro-morphology greatly changed (close to dentinal exposure of large surfaces) or dentin surface exposed by ≤ 1/3
4	Enamel surface as described in grades 1, 2 and 3. Dentin surface exposed by > 1/3 or pulp visible through the dentin

This scale was used for grading severity of dental erosion via intra-oral photographs

**Table 3:** Ordinal scale used for grading cuppings on occlusal surfaces of mandibular first permanent molars (Hasselkvist et al. 2010)

Grade	Criteria
0	No cupping/intact cusp tip
1	Rounded cusp tip^a^
2	Cupping ≤ 1 mm
3	Cupping > 1 mm
4	Fused cuppings: at least two cuppings are fused together on the same tooth

^a^Changed morphology compared to the assumed original anatomy at the time of eruption. This scale was used for grading cuppings via intra-oral photographs

All colour photographs were examined by two calibrated specialist Paediatric dentists (MSJ, KC) at the Department of Paediatric Dentistry, Eastman Institute, Stockholm.

### Calibration

Two of the authors at the Department of Paediatric Dentistry who were going to grade DE via the intra-oral photographs were calibrated prior to the grading. Each dentist examined the same photographs from 38 adolescents (228 surfaces). In case of any uncertainty about the severity of the lesions the lower score was selected. To establish the intra-examiner agreement, the photographs were re-examined 14–21 days after the initial examination. The third examination was performed by the two dentists together in order to determine inter-examination agreement. The mean inter-examiner reliability on a surface-by-surface basis on photographs was 0.50 (Kw), indicating moderate agreement, while the mean intra-examiner was 0.91(Kw), indicating a very good level of agreement (Altman 1995). To improve the inter-examiner agreement before the study the dentists examined photographs from the first 20 patients once again and in case of disagreement compared the erosions on photographs with graded dental erosion according to the literature (Johansson and Carlsson 2006).

### Statistical analysis

Sample size calculation, with 90% power and a 5% significance level, resulted in a minimum of 900 patients, to detect a 10% difference in erosion between the three clinics in pair wise comparisons (3 × 300 = 900). The calculation was based on 20% prevalence of DE (Hasselkvist et al. 2010).

The distribution of DE by clinics and age groups (15 vs. 17 years) were compared using the Chi square test. Differences between the two age groups and between groups with different background factors for DE were calculated and considered significant at p < 0.05 (Table 4).

**Table 4:** Bivariate logistic regression predicting dental erosion

Factor	Beta	SE	p	OR (CI)
Age (15 vs 17 years)	0.281	0.132	0.034	1.32 (1.30, 1.35)
Male gender	0.285	0.132	0.031	1.33 (1.31, 1.35)
Tooth hypersensitivity	0.376	0.150	0.012	1.46 (1.42, 1.49)
Frequency of soft drinks (3 categories^a^)			< 0.001	
Frequency of juice (3 categories^a^)			NS	
Juice at breakfast	0.183	0.132	NS	1.20 (1.18, 1.22)
Drinking after exercise	0.553	0.170	0.006	1.74 (1.69, 1.79)
Frequency of fruits > 3 times/day	0.347	0.156	0.027	1.41 (1.38, 1.45)
Frequency of apples > 3 times/day	0.561	0.243	0.021	1.75 (1.65, 1.86)
Frequency of citrus > 3 times/day	0.091	0.251	NS	1.10 (1.08, 1.17)
Time at computer (3 categories^a^)			NS	
Reflux	1.04	0.264	< 0.001	2.84 (2.64, 3.03)
Natrium Fluoride rinse	0.518	0.319	NS	1.68 (1.52, 1.86)
Frequency of tooth brushing (3 categories^a^)			NS	
Tooth brushing adjacent to breakfast -				
(3 categories^a^)			NS	
Frequency of chewing gum (3 categories^a^)			NS	
Mouthwash	− 0.085	0.164	NS	0.98 (0.89, 0.94)
Mouthwash type (2 categories^a^)			NS	

*Beta* regression coefficient, *SE* standard error, *P* probability value, *CI* confidence interval (95%)

^a^Questionnaire, Table 1

Data analyses were carried out using Statistical Package for the Social Science (SPSS, Version 22). For continuous variables, the mean and standard deviation (SD) were calculated. One-way analysis of variance (ANOVA) and an independent *t* test were used to compare the mean values on erosion. For categorical variables, a bivariate analysis of associations between the categorical variables on erosion was compared using the Chi Square test.

The multivariate analysis was performed on factors with significant influence on ‘erosion’ in the bivariate analyses. Multiple logistic regression with a forward stepwise procedure was used to evaluate the factors of ‘independent’ importance, accounted for in the ‘multivariate’ table (Table 5). Bi-and multivariate logistic regression analysis was used to calculate the odds ratios (OR) and 95% confidence intervals (CI). The ORs with 95% CI were used as estimates of the effects.

**Table 5:** Multiple logistic regressions predicting dental erosion. Odds ratio (OR) and 95% confidence intervals (CI)

Factor	Beta	SE	p	OR	CI
Age 17 vs. 15 years	0.273	0.136	0.045	1.31	1.01–1.71
Freq.soft drinks not often vs. several times a week	0.487	0.136	< 0.001	1.63	1.25–2.13
Drinking after exercise	0.479	0.175	0.006	1.61	1.15–2.28
Reflux	1.009	0.268	< 0.001	2.74	1.62–4.64
Constant	− 1.296	0.122			

*Beta* regression coefficient, *SE* standard error, *P* probability value, *CI* confidence interval (95%)

## Results

### Prevalence of clinically diagnosed dental erosion

Out of 1071 adolescents studied, DE was registered in 336 individuals (31.4%). DE was more frequent among 17 year old where erosive wear was diagnosed in 189 (34.3%) adolescents compared to 147 (28.3%) in 15 year olds. Erosive wear was more common in males, 188 individuals (34.4%) showed DE and 148 (28.2%) in females. The highest frequency of DE was found in Sollentuna, with 177 adolescents (44.5%) followed by Brommaplan, 88 (25.9%) and Södertälje 71 (21.3%) individuals respectively.

### Clinically diagnosed dental erosion and risk factors

**Fig. 2:**
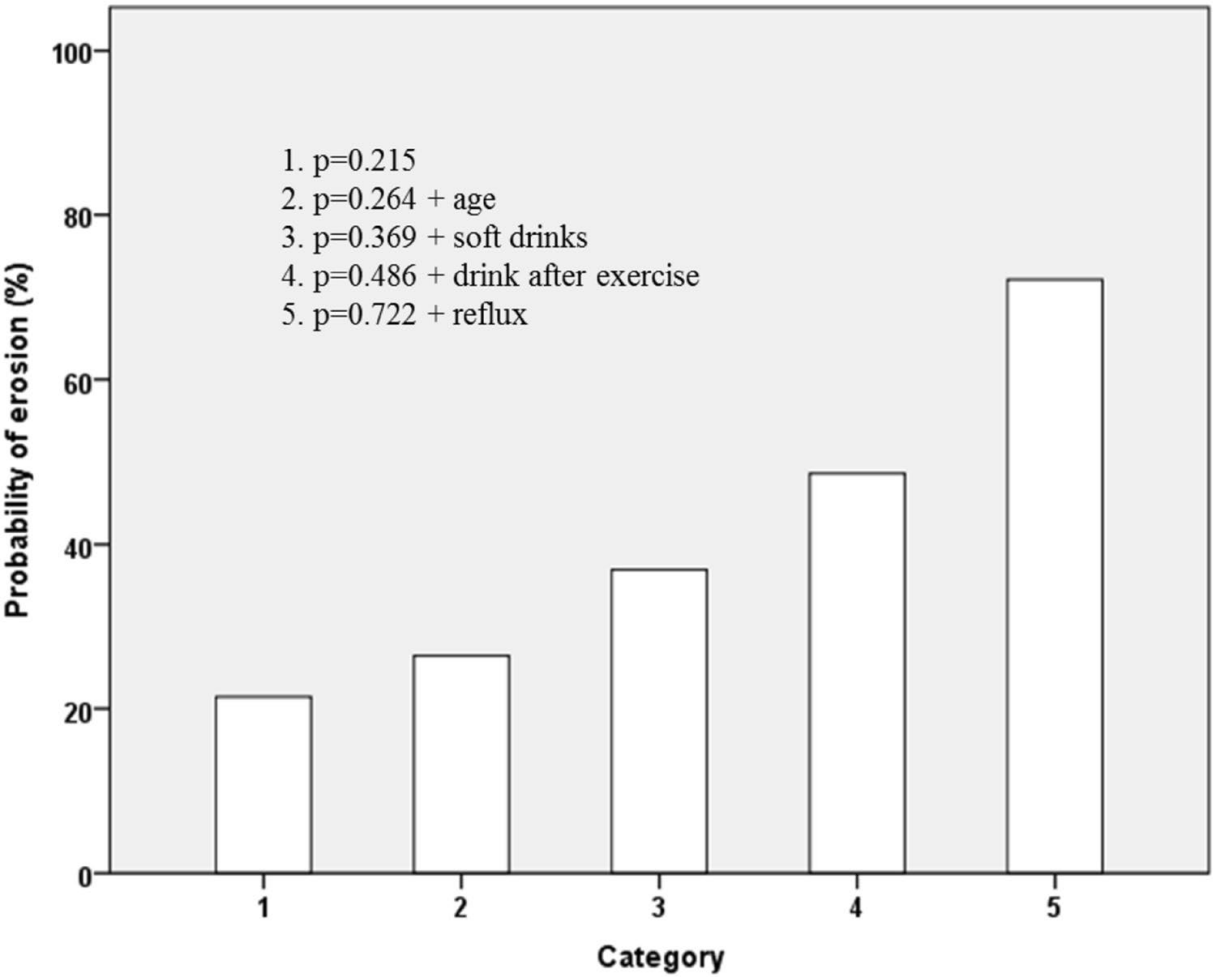
Cumulative percentage probability of dental erosion. 1. None of the predictors, identified in this study present; 2. age (17 years); 3. age + soft drinks several times a week; 4. age + soft drinks several times a week + juice or sport drink after exercise; 5. age + soft drinks several times a week + juice or sport drink after exercise + reflux

Consumption of soft drinks several times a week was more prevalent in individuals with DE than in those with no erosion (p < 0.001). The use of juice or soft drinks as a thirst quencher after exercise was also more common in adolescents with DE (p = 0.006). The occurrence of erosive wear was higher in individuals with tooth hypersensitivity (p = 0.012). Self-assessed gastric reflux was strongly associated with DE (p < 0.001)(Table 4). The probability of DE increased with the number of risk factors present, starting with 21.5% at baseline to 72.2% with all predictors identified for DE present (Fig. 2).

### Prevalence of dental erosion grades 3 and 4 in photographs

Among 1202 individuals erosive lesions grade 3 or 4 according to the photographs were registered in at least one of the four maxillary incisors or lower first molars among 220 adolescents representing one fifth of the adolescents. Males had a higher frequency of DE grades 3 and 4 with 136 individuals (22.3%) showing severe erosive wear compared to 84 females (14.4%) (p < 0.01). In addition, 17 year olds had higher frequencies of severe erosive wear with 134 individuals diagnosed with DE grade 3 and 4 (20.7%) compared to 86 (15.5%) 15 years old (p = 0.015).

## Discussion

In this study almost one-third of the group of the 15- and 17 year old adolescents in Stockholm County had erosive lesions and that the presence of DE could be linked to external risk factors such as soft drink and juice consumption and internal risk factors such as reflux and also to tooth hypersensitivity. These findings emphasise the need for a national epidemiological scoring system for DE as well as for linked preventive programmes to be implemented.

Adolescents from three areas in Stockholm County with a different social profile were examined in order to try to identify if DE was more common in certain social groups in society. Some 75% of the 15 and 17 years old in Stockholm County are patients in the Public Dental Service. This may probably indicate that adolescents visiting the Public Dental Service reflects the oral problems of the youth population in these areas.

The 15 and 17 years old adolescents were selected for the present study for several reasons. At these ages, selected marker teeth have been present in the mouth for approximately 9 and 11 years and thus exposed to erosive challenges. Further these ages are standard ages for regular dental health examinations in Sweden and they also represent groups when individual eating and drinking habits are changing from parenthood to more independence. The 17 year old adolescents have a more established free individual lifestyle reflecting social factors, dietary habits etc. Finally there is a higher probability of finding erosive lesions only in adolescents, as attrition and abrasion are less common compared with older individuals (Van’t Spijker et al. 2009).

Epidemiological studies on DE have shown a wide range of prevalence (Jaeggi and Lussi 2006).

In the present study, 31.4% of the individuals showed signs of erosive wear, 28.3% of the 15 years old and 34.3% of the 17 years old. Arnadottir et al. (2010) concluded that DE was seen in approximately 31% of 15 years old and in the systematic review by Salas et al. (2015a) the estimated prevalence of tooth erosion in children and adolescents 8–19 years was 30.4%. Mulic et al. (2013) found erosive wear in 38% among 18 years old. These studies represent various diagnostic methods and different age groups and countries. The frequency range of DE is in accordance with the present study.

Severe erosive wear (DE grades 3 and 4) was found in 18.3% of the individuals based upon the intra-oral photographs in the present study. That was consistent with 18% extensive erosion that Isaksson et al. (2013) found among 20 years old and in another Swedish study by Hasselkvist et al. (2010) where the corresponding figure for 18 and 19 years old was 22.3%. Different ages, groups studied and/or scoring systems used can explain the differences in prevalence between these studies. One a specific scoring method for measurement of erosive wear, clear in criteria and reproducible, is needed. Various scales and scoring systems in different studies created problems when comparing the prevalence figures (Huysmans et al. 2011; Milosevic 2011).

In the present study DE was more common in 17 year olds (34.3%) than in 15 year olds (28.3%). Severe erosive wear (grades 3 and 4) was also more prevalent in 17 year olds (20.7%) compared to 15 year olds (15.5%). Longitudinal studies conducted by Dugmore and Rock (2003) and El Aidi et al. (2010), reported rising prevalence rates with increasing age. Hasselkvist et al. (2016) found that progression of erosive lesions in Swedish adolescents aged 13–14 years followed to age 17–18 years was common and related to lifestyle factors. These findings support the present study showing that DE is more frequent and more severe in the group of 17 year olds.

Males had significantly more erosive wear (34.4%) than females (28.2%) in the present study. Severe DE among males was 22.3% compared to females 14.4%, based upon intra-oral photographs. Gender differences regarding presence and severity of DE, showing that males had higher prevalence figures and more lesions into dentine than females, has been reported previously (Dugmore and Rock 2003; Hasselkvist et al. 2010, 2014; Mulic et al. 2013).

Many studies have confirmed an association between DE and soft drink consumption among adolescents (Johansson et al. 2002; Dugmore and Rock 2004b; Jensdottir et al. 2004; Mulic et al. 2012; Isaksson et al. 2013; Hasselkvist et al. 2014; Muller-Bolla et al. 2015; Salas et al. 2015b). These results corresponded with the results of the present study where DE was more common in subjects drinking soft drinks several times a week. In the present study, we also found an association between erosive wear and drinking juice or sport drinks after exercise thus indicating that erosive wear can be linked to the lifestyle of young people. This was in line with data reported by Myklebust et al. (2003) in which consumption of juices and soft drinks during exercise appeared to be part of the aetiology of DE in young recruits. Further, Frese et al. (2015) found that endurance training resulted an increased risk for DE caused by a decrease in salivary flow rates during maximum workload.

The estimated probability for DE in this study was 21.5% even though none of the predictors for DE were present (Fig. 2). Other factors that may influence the presence of DE are e.g. individual susceptibility, differences in the enamel composition due to polymorphisms in enamel formation genes, variation in the pellicle, and variation in the saliva content (Schlueter and Tveit 2014; Sövik et al. 2015; Uhlen et al. 2016). Also, other acidic sources than the ones identified in the present study questionnaire may have existed.

Tooth hypersensitivity was more common in adolescents with DE. This finding was consistent with Shitsuka et al. (2015) showing that the presence of DE was associated with the occurrence of dentine hypersensitivity in children. The results were also in line with Olley et al. (2015) reporting that tooth wear, predominately active erosive wear, was important in the aetiology of dentine hypersensitivity on occlusal/incisal tooth surfaces. Furthermore, dentine hypersensitivity is more likely initiated if subjects had consumed acidic beverages more recently. Persons with regular or abusive consumption of soft drinks, persons living on a special diet, and persons suffering from endogenous erosion are at particular risk for the development of severe DE and for the development of hypersensitivity. Adequate prophylaxis for DE will prevent hypersensitivity but sometimes restorative therapy may be necessary to relieve these problems (Schlueter et al. 2012; Peutzfeldt et al. 2014).

Self-assessed gastric reflux was a factor strongly associated with erosive wear in this study. The relationship between DE and GERD has previously been reported in different studies (Holbrook et al. 2009; Mulic et al. 2012; Tantbirojn et al. 2012; Wilder-Smith et al. 2015). The dentist may be the first to observe signs of ‘silent reflux’ or an eating disorder in the form of DE (Ranjitkar et al. 2012; Johansson et al. 2012b). Eating disorders are increasing in contemporary society and younger people are affected, highlighting how important it is for the dentist to be aware of these problems, and to be able to diagnose early erosion in children and adolescents (Campbell and Peebles, 2014).

The present study was conducted at three dental clinics belonging to the Public Dental Service in Stockholm County Council and from different social areas but it was not possible to link DE significantly to adolescents from a certain social group. One possible explanation can be that Södertälje had a higher number of incomplete registrations (n = 92, 70%) than the other two clinics. Previous studies have shown no consensus regarding erosive wear and socio-economics (Al-Dlaigan et al. 2001; Bardsley et al. 2004; El Aidi et al. 2010).

The present study emphasises the importance of early diagnosis of DE to avoid drink hypersensitivity and restorative procedures. Furthermore, it highlights the need for a thorough medical history to clarify both he exogenous and endogenous factors that may influence disease progression. Dental health education and motivation to bring about a lifestyle change is necessary as well as collaboration with medical colleagues in reflux diseases and eating disorders to investigate the causes and to prevent progression of existing lesions.

The present study stresses the importance of registration of erosive wear at the regular dental health examination and to agree on a national and international epidemiologic scoring system. Future studies of dental erosive wear and preventive measures are needed, as there is still a lack of knowledge.

Clinical implication: Clinicians are recommended to focus on this problem to diagnose at an early stage and also to investigate the cause of DE that often can be explained by exogenous factors such as a frequent consumption of acidic drinks but also endogenous causes such as reflux disease and eating disorders. A close collaboration with medical professionals in these areas is essential for referral of patients. Appropriate preventive and restorative measures must also be provided depending on the severity of erosion damage and check ups on a regular basis to avoid further loss of tooth material.

## Conclusions

The present study indicates that dental erosions is a common problem among adolescents of today and are linked to both internal and external risk factors. The present study also emphasises the importance of early diagnosis.
